# Astrocytes in the nucleus of the solitary tract: Contributions to neural circuits controlling physiology

**DOI:** 10.1016/j.physbeh.2020.112982

**Published:** 2020-09-01

**Authors:** Alastair J. MacDonald, Kate L.J. Ellacott

**Affiliations:** Institute of Biomedical & Clinical Sciences, University of Exeter Medical School, Level 4, RILD, Barrack Rd, Exeter EX2 5DW, UK

**Keywords:** Astrocyte, Autonomic, Brainstem, Feeding, Glucose, Glutamate

## Abstract

The nucleus of the solitary tract (NTS) is the primary brainstem centre for the integration of physiological information from the periphery transmitted via the vagus nerve. In turn, the NTS feeds into downstream circuits regulating physiological parameters. Astrocytes are glial cells which have key roles in maintaining CNS tissue homeostasis and regulating neuronal communication. Recently an increasing number of studies have implicated astrocytes in the regulation of synaptic transmission and physiology. This review aims to highlight evidence for a role for astrocytes in the functions of the NTS. Astrocytes maintain and modulate NTS synaptic transmission contributing to the control of diverse physiological systems namely cardiovascular, respiratory, glucoregulatory, and gastrointestinal. In addition, it appears these cells may have a role in central control of feeding behaviour. As such these cells are a key component of signal processing and physiological control by the NTS.

## 1 Introduction

The nucleus of the solitary tract (NTS) is a major interoceptive hub in the brainstem which forms part of the dorsal vagal complex (DVC) along with the area postrema (AP) and dorsal motor nucleus of the vagus (DMX). The vagus nerve (10th cranial nerve) innervates most of the major internal organs including the heart, lungs and gastrointestinal tract, and its afferent branch sends terminals to the NTS via a nerve bundle called the solitary tract (ST). The majority of the inputs from the vagus terminate in the caudal NTS while more rostral areas are innervated by the facial nerve (7th cranial nerve) and glossopharyngeal nerve (9th cranial nerve). Vagal afferents form glutamatergic synapses onto second-order neurons relaying information from the periphery to the NTS [1,2]. NTS neurons project locally to preganglionic motor neurons in the DMX and to other sites in the brainstem, midbrain and hypothalamus to drive appropriate physiological responses to incoming signals [3]. To this end, the NTS has been shown to be the point of origin for central nervous system (CNS) processing of cardiovascular, respiratory, glucoregulatory and satiety signalling [4], [5], [6], [7]. Much of this work to date has focussed on neurons, but a growing number of studies are examining roles for non-neuronal cells in the synaptic and physiological processes of the NTS.

Astrocytes are glial cells that populate the entire CNS. These cells are crucial to brain function, providing structural and metabolic support to neurons and playing roles in synaptic transmission and cerebrovascular coupling (for review see [8]). Astrocytes predominantly signal via fluctuations in intracellular Ca^2+^ coupled to gliotransmitter release [9,10]. These astrocytic Ca^2+^ variations have been demonstrated in response to neurotransmitters and lead to alterations in downstream signalling, including release of gliotransmitters (e.g. glutamate, ATP) and modulation of glutamate transport [10,11]. Astrocytes in a variety of different brain regions are implicated in the control of a wide range of neural systems including memory [12,13], addiction [14], [15], [16] and, of importance to this review, autonomic control [17,18] and the regulation of feeding behaviour [19], [20], [21], [22], [23], [24], [25]. The NTS is no exception, and here we highlight research showing that astrocytes in this brain region support and contribute to neural circuits controlling physiology.

## 2 Astrocytes modulate synaptic transmission in the NTS

**Fig. 1:**
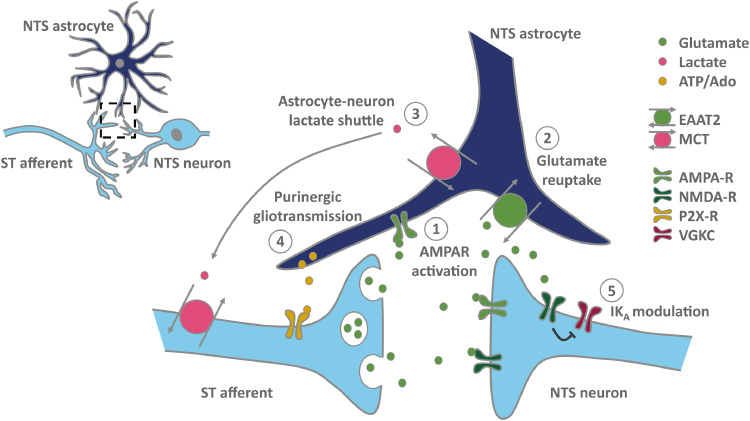
A simplified schematic of astrocyte modulation of synaptic transmission in the NTS. 1) Astrocytes respond to synaptic glutamate *via* AMPA-receptors (AMPA-R) expressed on the cell surface; 2) Astrocytes clear glutamate from the synapse to restrain neuronal firing and maintain presynaptic glutamate levels *via* EAAT2; 3) Astrocytes provide fuel to neurons in the form of lactate in order to maintain fidelity of synaptic transmission; 4) Astrocytes provide tonic modulation to synaptic transmission in the form of purinergic gliotransmission (release of ATP which may be converted to adenosine in the synaptic cleft) and 5) altering post-synaptic excitability by modulating presence of the a-type potassium current (IK_A_) which restrains action potential firing. Abbreviations: Ado = adenosine, ATP = adenosine triphosphate, EAAT2 = excitatory amino acid transporter 2, MCT = monocarboxylate transporter, NTS= nucleus of the solitary tract, ST = solitary tract, VGKC = voltage gated potassium channel.

Synaptic transmission between vagal afferent terminals and second-order viscerosensory neurons allows for the appropriate autonomic and behavioural response to physiological challenges. Vagal afferents entering the NTS via the ST release glutamate onto postsynaptic neurons. This can be modelled experimentally where in *ex vivo* brain slices, electrical stimulation of the ST produces short-latency, glutamate-mediated, excitatory postsynaptic currents (EPSCs) in second-order NTS neurons [1,2]. Astrocytes ensheathe glutamatergic synapses in the NTS providing structural evidence for a role in synaptic transmission [26] and indeed, a number of functional studies (described below) have demonstrated that astrocytes are critically involved in this process (Fig. 1).

NTS astrocytes express Ca^2+^ permeable AMPA receptors (AMPA-R). Using Ca^2+^ imaging from astrocytes and neurons in *ex vivo* NTS rat brain slices, McDougal and colleagues demonstrated that astrocytes can detect glutamate released following electrical ST stimulation and respond with increases in intracellular Ca^2+^, both from extra- and intracellular sources [27]. This ST stimulation evokes activation of AMPA-R on astrocytes allowing Ca^2+^ influx to the cell, which drives Ca^2+^-induced Ca^2+^-release further increasing Ca^2+^ levels via liberation from intracellular stores. These Ca^2+^ elevations are sensitive to the AMPA-R antagonist NBQX but not antagonists for metabotropic glutamate receptors (mGlu-R) 1 or 5 nor NMDA receptors (NMDA-R) [27]. Further supporting a role for astrocytic AMPA-R in integrating information encoded by the ST, in a separate study ST stimulation evoked time-locked, inward currents in astrocytes that were abolished by the AMPA-R antagonist DNQX [28]. The ionic flow contributing to this inward current was not directly assessed. Thus, NTS astrocytes are sensitive to incoming signals from the periphery via vagal afferent glutamate detection.

In addition to sensing ST-derived glutamate, astrocytes also contribute to transmission across the ST-NTS neuron synapse. Astrocyte function can be selectively inhibited with fluoroacetate (FAC) and its metabolite fluorocitrate (FC). FC inhibits the tricarboxylic acid (TCA) cycle and both FC and FAC preferentially affect astrocytes at nanomolar concentrations used in experimental settings [29], [30], [31]. This is thought to be due to the tendency of astrocytes and not neurons to take up and utilize acetate for cellular metabolism [32,33]. A second mechanism of action may be due to buffering of intracellular Ca^2+^ due to accumulation of citrate, a Ca^2+^ chelator [34], [35], [36]. In brain slices from rats, metabotropic receptor dependent Ca^2+^-signalling and gliotransmission in NTS astrocytes has been demonstrably and reversibly inhibited by 30 minute treatment of FC followed by a 10 minute washout period [37].

In the presence of FAC, both ST-evoked EPSC amplitude and spontaneous EPSC frequency are reduced in NTS neurons that project to the ventrolateral medulla (VLM) [28]. This tonic, astrocytic contribution to synaptic transmission is likely mediated by release of ATP since antagonism of P2X purinergic receptors with the broad-spectrum ligand iso-PPADS recapitulates the effect of FAC, but the two do not have additive effects [28]. Furthermore, an increase in extracellular ATP observed in this study following ST-stimulation was reduced by FAC. Evidence also suggests that astrocytes can restrain neuronal excitability directly in NTS neurons; astrocyte inhibition with FAC reduces the NTS neuronal A-type potassium current (IK_A_) [38]. Under normal conditions, this outward potassium current present at the beginning of the initial depolarisation acts to restrain neuronal action potential threshold and firing. While the underlying mechanism has not yet been fully explored in the NTS, in the hypothalamus IK_A_ is inhibited when astrocytic glutamate uptake is reduced due to activation of extra-synaptic NMDA-Rs [39]. In postsynaptic neurons, ST-evoked AMPA EPSC amplitude is lower from brain slices in the presence of FAC while ST-evoked NMDA EPSC amplitude is greater [40]. This demonstrates that astrocytes provide tonic neuromodulation which has inverse effects on signalling via the two main types of ionotropic glutamate receptor.

A second mechanism by which astrocytes may support ST-NTS neurotransmission is by active supply of the metabolite lactate [41]. It has been theorised that in the brain, glucose is the main fuel source for astrocytes which then metabolise this to lactate. Astrocyte-derived lactate is then shuttled via monocarboyxlate transporters (MCTs) to neurons where it is metabolised to pyruvate and used to fuel the TCA cycle [42,43]. However this hypothesis, termed the astrocyte-neuron lactate shuttle, is controversial [44] and remains a topic of debate [45,46]. When MCTs are pharmacologically inhibited with 4-CIN, phloretin or D-lactate in rat NTS brain slices, the amplitude of ST-evoked neuronal EPSCs are reduced. Since this effect is rescued by providing extracellular lactate it was concluded that ST-NTS synaptic fidelity is reliant on lactate from astrocytes transported through MCTs on astrocytes and neurons [41].

A further mechanism by which astrocytes may modulate ST-NTS neurotransmission is by regulating synaptic glutamate availability. Astrocytes clear glutamate from the synaptic cleft and recycle it to neurons in the form of glutamine, which can then be used for further glutamate synthesis by neurons [47]. Excitatory amino acid transporter 1 (EAAT1) and EAAT2, are expressed primarily by astrocytes [48]. In the NTS this astrocytic glutamate reuptake appears to be essential for normal synaptic function [49], [50], [51]. Pharmacological blockade of both EAAT1 and EAAT2 with DL-*threo*-β-benzyloxyaspartic acid (TBOA) elevates synaptic glutamate as evidenced by NTS neuronal depolarisation and action potential firing, and increased spontaneous EPSCs [49]. Furthermore in NTS neurons, ST-evoked EPSCs are reduced by TBOA suggesting released glutamate is not being returned to the presynaptic terminal [49]. These effects can be recapitulated by dihydrokainate (DHK), an EAAT2 blocker [50,51]. In combination with immunohistochemical evidence [26], this indicates that EAAT2 is the primary glutamate transporter responsible for glutamate reuptake and recycling at the ST-NTS synapse [50,51]. Glutamate transporters on astrocytes appear to be pH sensitive since synaptic glutamate accumulates at pH 7.0, suggesting they may play an additional chemosensory role [52]. Given that astrocytes in more ventral chemosensory brainstem areas, including the retrotrapezoid nucleus, are directly pH sensitive and involved, in concert with local neurons, in detection of increasing PCO_2_ this may be a common feature of astrocytes that allows for chemodetection of low pH [53,54].

Thus, combined evidence from a number of independent research groups shows that astrocytes support synaptic transmission in the NTS by buffering and recycling synaptic glutamate [49], [50], [51], shuttling lactate to neurons [41] and providing tonic purinergic neuromodulation [28,38] (Fig. 1). Interestingly, they are also able to directly detect vagal glutamate release [27]. This raises the possibility that astrocytes modulate their functions in response to increased vagal input making them key integrators of information at this important integrative site.

## 3 Physiological and environmental stimuli modulate the morphology of NTS astrocytes

Astrocytes show regional variations in the expression of the cytoskeletal glial fibrillary acidic protein (GFAP), which is dynamically regulated in response to local variations in the brain microenvironment [55], [56], [57]. For example cortical astrocytes show low basal GFAP expression but dramatically upregulate this protein in response to tissue injury, suggesting that this change serves a reactive and possibly neuroprotective role [58]. Since GFAP forms intermediate filaments that make up the astrocyte cytoskeleton, an increase in GFAP expression results in altered morphology and increased branching of the cells. Precisely how this morphological change relates to cell function is still unclear, but it may allow dynamic ensheathment of synapses. Broadly speaking, high GFAP expression and branched morphology is considered indicative of astrocyte activation. Relatively, the NTS in rats has been described to have higher GFAP expression than other brainstem nuclei [59]. However, in contrast, some studies in mice observed lower levels of GFAP expression when compared with the rat example [60,61]. It is unclear if this represents a true species difference or is indicative of variations in the experimental conditions since GFAP expression in the NTS is highly dynamic (discussed below). Notably in rats, within the NTS the astrocytes are smaller, have a simpler morphology, and greater overlapping domains than other brainstem autonomic nuclei [62]. Since GFAP is not a uniformly expressed astrocyte marker [55,56] and is dynamically regulated [57,58], some studies have utilized other markers, including the calcium binding protein S100b [27].

Several studies have reported that NTS GFAP expression, commonly measured using immunoreactivity, is modulated by different experimental stimuli (Table 1). For example, inspiration of ozone gas increases vascular endothelial growth factor (VEGF) expression in NTS astrocytes and increases the branching of the cells [63], indicating the responsivity of these cells to respiratory challenge. Ozone inhalation also increases the astrocytic ensheathment of synapses in the NTS, which may represent a mechanism by which astrocytes regulate neuronal synaptic activity as a compensatory response to the physiological change [64]. GFAP is also increased in the NTS under conditions of hypoxia, at least during initial 24h [60,65,66]. Interestingly, blockade of microglial activation with minocycline decreases hypoxia-induced GFAP changes, indicating crosstalk between these two glial cell types in the NTS [60]. Taken together this suggests that NTS astrocytes are responsive to low oxygen and may be involved in mediating the central response to hypoxia (see Section 4.1).

**Table 1:** Summary of studies which have examined impact of experimental manipulations on NTS astrocyte immunoreactivity and/or morphology.

Stimulus	Outcome	Species	Reference
Ozone inhalation (3 hours)	Greater VEGF expression in NTS astrocytes than normoxic controls, increased branching of cells	Rat	Araneda *et al* 2008 [63]
Ozone inhalation (24 hours)	Greater glial coverage of synapses in NTS compared with untreated controls	Rat	Chounlamountry *et al* 2015 [64]
10% Oxygen inhalation	Greater NTS GFAP immunoreactivity (1 and 6 hours, compared with normoxic controls). 6 hour blocked by minocycline	Mouse	Tadmouri *et al* 2014 [60]
10% Oxygen inhalation	Greater GFAP immunoreactivity in NTS (4 and 24 hours, compared with normoxic controls), blocked by minocycline	Rat	Stokes *et al* 2017 [65]
10% Oxygen inhalation	Greater GFAP immunoreactivity in NTS after 10 days compared with normoxic controls	Rat	De La Zerda *et al* 2018 [66]
Thioacetamine injection (liver damage model)	Greater number of GFAP expressing cells in NTS compared with vehicle injected controls	Rat	Tsai *et al* 2017 [67]
Lateral ventricle STZ treatment (neurodegeneration model)	Greater S100b immunoreactivity in commissural NTS compared with vehicle injected controls	Rat	Ebel *et al* 2017 [68]
Intrastriatal 6-OHDA injection (Parkinsonian model)	Lower GFAP immunoreactivity in NTS after 60 days compared with shorter durations	Rat	Fernandes-Junior *et al* 2018 [69]
Two-kidney 1-clip hypertension	Greater number of GFAP-positive pixels in NTS than in normotensive controls	Rat	Melo *et al* 2019 [70]
Isoproterenol induced water drinking	No difference in GFAP immunoreactivity in NTS	Rat	Hardy *et al* 2018 [71]
Age	Greater GFAP immunoreactivity in NTS of aged (24 months) compared with young (6 months)	Rat	Hardy *et al* 2018 [71]
Prenatal (G11 or G16) X-irradiation	Greater number of GFAP expressing cells in NTS at P7-14 for G11 irradiated pups	Rat	Jacquin *et al* 2000 [73]
Prenatal cigarette smoke inhalation	No difference in GFAP immunoreactivity in NTS compared with non-exposed pups	Mouse	Machaalani *et al* 2019 [74]
Sudden infant death syndrome	Greater GFAP-positive cell density in NTS from SIDS victims than age-matched controls	Human	Biondo *et al* 2004 [75]
Chronic morphine treatment	Greater GFAP-positive cell density in NTS than vehicle treated, blocked by yohimbine treatment	Rat	Alonso *et al* 2007 [76]
Agouti related peptide neuron ablation	Greater number of GFAP-positive pixels in NTS than control mice	Mouse	Wu *et al* 2008 [77]
DVC tumour necrosis factor-α treatment	Greater c-FOS expression by NTS astrocytes than vehicle injected controls	Rat	Hermann and Rogers 2009 [78]
Unilateral chorda tympani nerve crush	Greater GFAP immunoreactivity in rostral NTS compared with uninjured controls or intact side	Mouse	Bartel 2012 [79]
12-hour high-fat chow intake	Greater GFAP immunoreactivity and branching profile than standard chow fed controls	Mouse	MacDonald *et al* 2020 [80]

In addition to hypoxic challenge, NTS GFAP expression is regulated in a diverse range of disease models associated with inflammation (Table 1). For example, NTS GFAP immunoreactivity is increased in a pharmacological rat model of liver failure, alongside impaired baroreflex sensitivity in these animals [67]. In a model where streptozotocin (STZ) is infused into the lateral ventricle of rats to induce neurodegeneration, greater s100b-immunoreactivity is seen in the NTS, accompanied by an impaired ability to increase breathing rate to compensate for hypoxia [68]. In another neurodegenerative disease model, 60 days after induction of Parkinsonian neuropathology (following striatal 6-hydroxydopamine injection) rats show decreased NTS GFAP immunoreactivity, which also correlates with respiratory changes [69]. Finally, in a 2-kidney 1-clip rat model where blood supply to one kidney is partially blocked to activate the renin-angiotensin-aldosterone system and produce hypertension, a greater number GFAP immunoreactive cells are observed in the NTS when compared with normotensive controls [70]. Importantly, NTS GFAP immunoreactivity increases with age in rats, which may in future be shown to associate with age-related changes in physiology in some cases [71]. Correlational evidence does not reveal whether this observed astrocyte plasticity is a cause, consequence or unrelated to the physiological phenotypes displayed by these models.

Astrocytes and their progenitors, radial glia, play a critical role in brain development [72] so it is noteworthy that NTS astrocyte morphology is also regulated by environmental stimuli presented during development. In rats exposed to prenatal X-ray irradiation at a critical period during development (gestational day 11 but not 16) greater GFAP immunoreactivity is observed in the NTS on postnatal days 7-14 [73], indicative of a prolonged astrocytic response to a single neonatal insult. In contrast, prenatal exposure of mice to cigarette smoke for the entire duration of gestation does not alter GFAP expression when compared to control mice [74], suggestive of potential compensatory adaptations following chronic exposure to stimuli or alternatively context specific regulation of NTS GFAP-expression .

In addition to those outlined above, a number of other studies have shown dynamic regulation of NTS astrocytes [75], [76], [77], [78], [79], [80]. While not discussed here, these are summarised in Table 1. Thus, taken together these studies show that GFAP expression is highly plastic in the NTS and is regulated by a myriad of stimuli. Future studies are needed to address the physiological consequences of this GFAP regulation, if/how NTS astrocytes differentiate between stimuli of different modalities, and how this mechanistically relates to other astrocyte functions.

## 4 Regulation of physiology by NTS astrocytes

### 4.1 Cardiorespiratory physiology

The NTS is the initial CNS detector of cardiovascular parameters (for review see [5,81]). Specifically, peripheral baroreceptors in the aortic arch detect increases in arterial pressure and increase vagal afferent input to the NTS. This NTS signal drives a corresponding decrease in heart rate and arterial pressure. This process is known as the baroreflex. The NTS also receives input from peripheral chemosensors which monitor blood O_2_ allowing for compensatory homeostatic chemoreflex responses to deviations in O_2_ or CO_2_ [81].

As discussed above, hypertension (in a rat model) influences expression of GFAP in the NTS [70], suggesting an astrocytic component to the physiological response. Indeed, functional work involving pharmacological ablation of NTS astrocytes has shown this to be the case. Saporins are toxic agents that, in their unconjugated form, selectively kill astrocytes when delivered to the rat NTS [82]. This ablation has severe consequences for cardiovascular function: lowering of 1) baroreflex sensitivity, 2) cardiopulmonary reflex sensitivity and 3) chemoreflex sensitivity. This suggests loss of correct physiological integration of peripheral cues by the NTS in these animals [82]. Rats with NTS astrocyte ablation show large variations in arterial pressure also indicative of poor cardiovascular reflex control. Critically, these rats show damage to their myocardium and in some cases die due to sudden cardiac arrest [82]. This failure of central cardiovascular control appears to be mediated by a loss of glutamate sensitivity since cardiovascular responses to NTS delivery of glutamate receptor agonists, AMPA and NMDA, are attenuated in NTS saporin treated rats [83].

In line with their role in integrating activity at the ST-NTS synapse, glutamate reuptake and recycling by NTS astrocytes is crucial for cardiovascular function. Blockade of all EAATs (by TBOA) or only EAAT2 (by DHK) causes cardiac depression and reduced baroreflex response to phenylephrine in rats [49], [50], [51]. This effect is blocked by kynurenate (an ionotropic glutamate receptor antagonist) and NBQX (an AMPA-R antagonist) suggesting that under normal conditions NTS astrocytes sequester synaptic glutamate in order to regulate NTS neuronal activity and resulting output from DMX neurons to the cardiorespiratory system [50,51].

Under hypoxic conditions, chemoreflex responses are engaged which initiate compensatory increases in respiratory and heart rate. This effect persists even when animals are returned to normoxia, suggesting some central adaptation to the low oxygen environment [84]. In rats and mice, astrocytes of the NTS respond to the initial phase of hypoxia with an increase in GFAP expression within 1-24 hours, an effect which is mediated in part by an interaction with microglia [60,65]. However, this microglial component does not appear to contribute to the maintenance of adaptation to sustained hypoxia [65,66]. At the synaptic level, sustained hypoxia causes adaptations in the rat NTS: increased postsynaptic excitability mediated by decreased expression of IK_A_ and greater amplitude NMDA and AMPA currents evoked by ST stimulation [38,40]. Inhibition of astrocytes with FAC reduces IK_A_, reduces the amplitude of ST evoked AMPA currents and increases the amplitude of ST evoked NMDA currents. These effects were not observed in slices from rats maintained under sustained hypoxic conditions [38,40]. This suggests that reduced astrocyte modulation of synaptic transmission may be an adaptive mechanism to increase neuronal sensitivity and drive respiration in sustained hypoxia.

Together these studies illustrate the necessity for tight control of synaptic glutamate in the NTS to maintain cardiorespiratory function, and the importance of astrocytes in this process. Furthermore, the published studies on adaptation to hypoxia suggest that astrocytes adapt to compensate for changes in physiological need.

### 4.2 Glucose sensing and counter-regulatory response

Orchestrated in part by the brain, the counter-regulatory response to hypoglycaemia (CRR) is initiated when blood glucose falls below the normal euglycemic range and is a multifaceted hormonal and neuronal response to restore blood glucose. The hindbrain is a critical site of hypoglycaemia detection and is required to drive appropriate counter-regulatory responses (namely feeding and increases in blood glucose) [85,86]. In particular, catecholaminergic neurons in the NTS and the VLM have been shown to be responsive to glucoprivic challenge [87]. Injection of non-metabolizable glucose analogues into these nuclei drives feeding and hepatic glucose production [88]. These studies suggest that the NTS, in addition to the VLM, is a central site of hypoglycaemia sensing and involved in mediating the CRR [6,89]. Neuronal connections exist between these two regions suggesting their coordinated activity may be important for mediating CRR [28,90,91].

In rat brain slices, 40% of NTS astrocytes increase their intracellular Ca^2+^ in response to low glucose or the non-metabolizable glucose analogue 2-deoxyglucose (2-DG; a glucoprivic agent) indicating the low glucose-sensitivity of these cells [92]. This response precedes a similar response in neurons and Ca^2+^ increases in both cell types are diminished following treatment with FC in mouse brain slices [93]. Delivery of 2-DG directly into the 4^th^ ventricle ([4V]; which is immediately proximal to the AP/NTS) increases blood glucose in anaesthetised rats, indicating that local reductions in brainstem glucose level are sufficient to drive compensatory changes in glucose homeostasis [94]. This blood glucose elevation is blocked by general inhibition of NTS astrocyte activity using FC delivered to the 4V or more specifically by the A1 adenosine-receptor antagonist DPCPX. This indicates that astrocyte-derived adenosine is a component in the detection and response to this glucoprivic stimulus [94].

Of critical importance to the CRR are NTS catecholaminergic neurons, identified by their expression the enzyme tyrosine hydroxylase (NTS^TH^). In brain slices from mice, the Ca^2+^ response of NTS^TH^ neurons to 2-DG is abolished by pre-treatment with either FC or the broad spectrum P2 purinergic receptor antagonist suramin [93]. The glucose transporter GLUT2 is a proposed glucose sensing protein and accordingly blockade of GLUT2 with quercetin abolishes astrocyte Ca^2+^ responses to low extracellular glucose and 2-DG in rat brain slices [95]. It appears that this observed Ca^2+^ response to low extracellular glucose is downstream of protein lipase C, which indicates that GLUT2 may engage second messenger systems in addition to its transporter function [95]. Consequently, it appears that NTS astrocytes are glucose sensors that in hypoglycaemic conditions relay this signal to, and/or enhance intrinsic glucose sensitivity of NTS^TH^ neurons via purinergic gliotransmission in order to drive the appropriate CRR.

Expression of GLUT2 has been demonstrated on NTS astrocytes of the rat by electron microscopy [96]. In mice, brain wide deletion of glucose transporters abolishes the CRR. However, in these animals restoration of GLUT2 expression in astrocytes alone is sufficient to restore glucagon secretion and DVC c-FOS expression in response to a systemic injection of 2-DG [97]. This indicates that direct sensing of low glucose by astrocytes is sufficient to mount a CRR.

A study using a mouse model expressing the fluorescent protein td-tomato in GLUT2-expressing cells found labelling of intrinsically glucose-inhibited GABAergic neurons in the NTS [98]. In *ex vivo* NTS slices these neurons increase their membrane potential and excitability in response to low extracellular glucose. This effect is recapitulated by depletion of intracellular ATP by oligomycin or activation of AMP-activated protein kinase (AMPK) by AICAR. In addition, this effect is abolished by inhibition of AMPK with compound C [98]. This shows that depletion of intracellular ATP and subsequent activation of AMPK drives excitability in conditions of low glucose in these neurons. The sufficiency of GLUT2-mediated glucose transport for conferring low glucose-sensitivity was not investigated and indeed some td-tomato labelled neurons did not express detectable levels of GLUT2 by RT-PCR suggesting glucose-sensitivity of these neurons could be GLUT2-independent [98]. These neurons clearly form a key component of CRR-driving circuitry since their optogenetic activation increases glucagon secretion [98]. In support of this, chemogenetic activation of GABAergic NTS neurons increases hepatic glucose production in mice [99].

Thus, it appears that in concert with glucose-inhibited NTS neurons, astrocytes in the NTS are involved in sensing low glucose levels. In NTS slices from rats the intracellular Ca^2+^ rises in low-glucose responsive astrocytes precede those of low-glucose responsive neurons by 50 seconds on average and a similar relationship is seen in NTS slices from mice [92,93]. The temporal nature of this response in rodent brain slices suggests that NTS astrocytes are a primary detector of, and may enhance responses of NTS^TH^ neurons (and other glucose sensitive NTS neurons e.g. GABAergic GLUT2 expressing neurons) to low glucose, at least in this experimental configuration [92,93]. In glucose-inhibited NTS neurons the response to low glucose depends on depletion of intracellular ATP and subsequent activation of AMPK [98]. This is consistent with the reported cellular compartmentalisation of glucose metabolism in the brain (i.e. the astrocyte-neuron lactate shuttle, discussed in detail below). However, it is still debated whether the astrocyte-neuron lactate shuttle is a ‘rule’ for brain metabolism or merely one mechanism of glucose metabolism [44,46]. This raises the possibility that astrocyte-independent neuronal glucose sensing mechanisms also play a role, although experiments with FC suggest their contribution is not sufficient to drive CRR in anaesthetised rats [94].

Influenced by Rogers and Hermann [100], we propose a working model of low glucose detection: In conditions of low glucose, astrocytic GLUT2 increases PLC signalling leading to Ca^2+^ signalling in astrocytes [95]. This results in purinergic gliotransmission which may modulate the firing of neighbouring neurons [93,94]. Furthermore, when lower glucose is available for conversion to lactate in astrocytes (and subsequent shuttling to neurons) this may result in reduced neuronal ATP generation. In glucose-inhibited NTS neurons this causes AMPK-dependent enhanced excitability and increased glucagon and hepatic glucose production [98,99]. This is supported by evidence indicating that Ca^2+^ responses to extracellular low glucose or glucopivation are attenuated in glucose-inhibited NTS neurons and NTS^TH^ neurons in brain slices from mice [93]. In addition, some neurons expressing GLUT2 and glucose sensing enzymes such as glucokinase may be specialised for direct low glucose sensing independent of astrocytes, but the necessity of neuronal GLUT2 for low glucose detection in the NTS remains to be demonstrated [98,101,102].

While NTS astrocyte integration of glycaemic status appears important for the physiological response to low glucose, what remains to be determined is whether astrocyte glucose sensitivity is reduced after recurrent hypoglycaemia, which may lead to blunted CNS glucose sensitivity and attenuation of the CRR [103]. *In vitro,* human primary astrocytes show metabolic adaptations following recurrent exposure low glucose levels, which has been suggested reflect a compensatory response to preserve function [104]. As discussed above, under sustained hypoxia astrocytes contribute to long-term adaptive changes in the physiological response to this stimulus, raising the possibility that this may also be true in the context of hypoglycaemia.

### 4.3 Food intake and gastric motility

There is a large body of evidence implicating NTS neurons in the integration of viscerosensory signals from the stomach and gastrointestinal tract, including encoding of satiety and meal termination [7]. A role for astrocytes in this process has only recently begun to be investigated. Reiner and colleagues investigated astrocytes as components of the glucagon-like peptide 1 (GLP-1) signalling system. In the periphery GLP-1 is released from enteroendocrine cells to excite sensory vagal neurons which innervate the stomach and intestine (for review see [105]). While this peripheral GLP-1 is not thought to enter the brain as it is quickly degraded, the NTS contains a population of GLP-1 synthesising neurons (preproglucagon [PPG] neurons) and cells that express the GLP-1 receptor (GLP-1R) [61,106,107]. In rats, peripheral or 4V injection of a fluorescent GLP-1R agonist (fluoro-exendin-4) revealed binding to GLP-1Rs on both NTS neurons and astrocytes, an effect that is reduced by pre-treatment with a GLP-1R antagonist [108]. This is further supported by the finding that exendin-4 causes an increase in intracellular Ca^2+^ in 40% of NTS astrocytes in rat brain slices. Critically, NTS pre-treatment with FC abolished the inhibitory effect of exendin-4 directly delivered to the NTS on food intake. Taken together these data suggest that NTS astrocytes are a component of the central GLP-1 satiety system, although the molecular mechanism by which they exert their effects has not been fully investigated [108].

In addition to exendin-4, cultured rat brainstem astrocytes show increased intracellular Ca^2+^ in response to application of ghrelin and leptin suggesting that, at least *in vitro*, brainstem astrocytes respond to a diverse range of hormonal signals that regulate appetite [109]. Ghrelin and leptin are canonically considered as opposing signals so the observation that both yield the same response in cultured brainstem astrocytes is of interest. The precise nature of Ca^2+^ signal diversity in astrocytes is beginning to be understood and may provide insight into how ‘opposing’ stimuli are processed within a single cell [10,110].

Although fluoro-exendin-4 binds to astrocytes in the rat NTS [108], observations from a transgenic mouse line expressing green fluorescent protein in GLP-1R expressing cells indicate no labelling of NTS astrocytes [61]. It remains to be demonstrated whether the contrasting results of Reiner *et al* and Cork *et al* reflect a species difference or are the result of two different methodological approaches to detect cells that express GLP-1R, but raises the possibility that astrocytes may play this important role in rats but not mice.

Further support for a key role of NTS astrocytes in the control of feeding comes from a series of studies on endozepines. NTS astrocytes (and tanycyte like cells of the area postrema known as vagliocytes [111]) express octadecaneuropeptide (ODN) [112] an endozepine cleaved from acyl-CoA-binding protein (ACBP) which acts to supress food intake [113]. Delivery of ODN into the 4V supresses food intake in rats and induces c-FOS expression in the NTS [112]. Thus, under normal conditions it is possible that NTS astrocytes and vagliocytes may secrete ODN to regulate food intake. Indeed, in the arcuate nucleus of the hypothalamus astrocytes release ACBP (cleaved extracellularly to ODN), which activates pro-opiomelanocortin neurons to supress appetite *via* its G-protein coupled receptor ODN-GPCR [114].

Work from our group indicates a role for NTS astrocytes in the control of food intake. We found that 12-hour consumption of a high-fat high-sucrose diet increases GFAP immunoreactivity and astrocyte process branching within the mouse NTS [80]. We also examined the consequence of NTS astrocyte activation on feeding behaviour using designer receptors exclusively activated by designer drugs (DREADDs). In mice expressing DREADDs in DVC astrocytes, stimulation of these cells with the ligand clozapine-N-oxide supresses nocturnal feeding and refeeding after a fast. This effect appears to be mediated by activation of neighbouring neurons since c-FOS expression was observed in the NTS and the lateral parabrachial nucleus (lPBN), a target a downstream of the NTS [80]. Activation of NTS astrocytes can also reduce gastric motility (discussed below) which may contribute to the observed hypophagia, although this was not measured in our study. While further work is needed, this indicates that NTS astrocytes sense caloric excess and gastric distention likely in part by sensing local neuronal activity and may contribute to driving a compensatory decrease in food intake. Due to the proximity of NTS astrocytes to the 4V/AP region the possibility of direct sensing of changes in circulating nutrients by astrocytes in this brain region cannot be excluded.

In addition to integration of information on satiety and meal termination, the NTS also contributes to modulation of parasympathetic tone to the gastrointestinal tract via preganglionic motor neurons in the DMX (for review see [115]). This tone can be influenced by astrocytes [37,116]. Astrocytes in the NTS express the protease-activated receptor 1 (PAR1) and activation of these receptors by 4V delivery of the agonist SFLLRN-NH2 reduces gastric motility and emptying in rats [116]. Activation of PAR1 on NTS astrocytes increased intracellular Ca^2+^ in neighbouring neurons via direct activation of NMDARs and by increasing presynaptic glutamate release [37]. The presynaptic effects of PAR1 activation appear to be mediated by transient receptor potential cation channel subfamily V member 1 (TRPV1) since they were absent in the presence of TRPV1 antagonists (capsazepine or SB366791) or in TRPV1 knock out rats [117]. Given that PAR1 is activated by serine proteases, including thrombin, it has been proposed that this system may be responsible for the autonomic dysfunction observed in patients suffering bleeding head injuries [116].

## 5 Conclusion

The studies reviewed here clearly illustrate the importance of NTS astrocytes in both effective synaptic transmission (Fig. 1) and physiological control. These cells sustain and regulate glutamatergic neurotransmission between ST afferents and second-order NTS neurons via a number of mechanisms: lactate shuttling, glutamate reuptake and purinergic gliotransmission. Furthermore, NTS astrocytes directly detect vagal glutamate which may allow activity-dependent regulation of these tonic functions. NTS astrocytes show dynamic changes in GFAP expression and cellular morphology in response to numerous environmental and experimental stimuli (Table 1). This is reflected in functional studies demonstrating their importance in cardiovascular, pulmonary, blood glucose, food intake and digestive control.

Further work is needed to elucidate the mechanisms by which astrocytes in the NTS can alter their synaptic support functions in an activity-dependent manner; for example, do astrocytes regulate EAAT2 glutamate transport in response to AMPA-R activation? Also, of interest is the integration of neuronal and hormonal cues by NTS astrocytes since they have been shown to respond to both. The application of cell-type specific genetic tools for cellular monitoring and manipulation [118] to NTS astrocytes will be critical to allow more detailed investigation of their contribution to synaptic and physiological functions.

## Declaration of Competing Interest

The authors of this manuscript declare no conflict of interest.
